# The first detection of *Anaplasma capra*, an emerging zoonotic *Anaplasma* sp., in erythrocytes

**DOI:** 10.1080/22221751.2021.1876532

**Published:** 2021-02-18

**Authors:** Yongshuai Peng, Chenyang Lu, Yaqun Yan, Ke Shi, Qian Chen, Cong Zhao, Rongjun Wang, Longxian Zhang, Fuchun Jian, Changshen Ning

**Affiliations:** a College of Veterinary Medicine, Henan University of Animal Husbandry and Economy, Zhengzhou, People’s Republic of China; b College of Veterinary Medicine, Henan Agricultural University, Zhengzhou, People’s Republic of China; c International Joint Research Laboratory for Zoonotic Diseases of Henan, Zhengzhou, People’s Republic of China

**Keywords:** *Anaplasma capra*, zoonotic, erythrocyte, tick-borne, *Rickettsia*

## Abstract

An emerging infectious disease caused by “*Anaplasma capra*” was reported in a 2015 survey of 477 hospital patients with a tick-bite history in China. However, the morphological characteristics and parasitic location of this pathogen are still unclear, and the pathogen has not been officially classified as a member of the genus *Anaplasma*. *Anaplasma capra*-positive blood samples were collected, blood cells separated, and DNA of whole blood cells, erythrocytes, and leukocytes extracted. Multiplex PCR detection assay was used to detect whole blood cell, erythrocytes and leukocytes, DNA samples, and PCR identification, nucleic acid sequencing, and phylogenetic analyses based on *A. capra groEL*, 16S rRNA, *gltA,* and *msp4* genes. Scanning electron microscopy (SEM), transmission electron microscopy (TEM), Wright–Giemsa staining, chromogenic in situ hybridization (CISH), immunocytochemistry, and indirect immunofluorescence assay (IFA) were used to identify the location and morphological characteristics of *A. capra*. Multiple gene loci results demonstrated that erythrocyte DNA samples were *A. capra*-positive, while leukocyte DNA samples were *A. capra*-negative. Phylogenetic analysis showed that *A. capra* is in the same clade with the *A. capra* sequence reported previously. SEM and TEM showed one or more pathogens internally or on the outer surface of erythrocytes. Giemsa staining, CISH, immunocytochemistry, and IFA indicated that erythrocytes were *A. capra*-positive. This study is the first to identify the novel zoonotic tick-borne *Anaplasma* sp., “*Anaplasma capra*,” in host erythrocytes. Based on our results, we suggest revision of Genus *Anaplasma* and formally name “*A. capra*” as *Anaplasma capra* sp. nov.

## Key points

- First identified the novel zoonotic tick-borne *Anaplasma* sp. in host erythrocytes.
- Suggest formally name the novel erythrocytes pathogen as *Anaplasma capra* sp. nov.

## Introduction

In 2015, an emerging infectious disease caused by “*Anaplasma capra*,” was reported in a survey of 477 hospital patients with tick-bite history in Mudanjiang Forestry Central Hospital, Heilongjiang Province, China. A total of 28 (6%) of the 477 patients were diagnosed with “*A. capra,*” and the pathogen was isolated from three cases [1]. Patients positive for this pathogen presented with an undifferentiated influenza-like illness, gastrointestinal symptoms, rash, eschar, regional lymphadenopathy with potential progression to central nervous system involvement, and cerebrospinal fluid pleocytosis [1]. *Anaplasma capra* was provisionally nominated as a novel tick-borne zoonotic *Anaplasma* sp. by Li et al. (2015) [1] after being first identified in a goat (*Capra aegagrus hircus*) [2]. *Anaplasma capra* has been shown to infect humans, ruminants (sheep, goat, cattle, etc.), pet dogs, and wild animals (e.g. takin, musk deer, goral) [1,3–8], and has been detected in a variety of ticks, e.g. *Ixodes persulcatus*, *Haemaphysalis longicornis*, *H. concinna*, *H. qinghaiensis,* and *Rhipicephalus microplus* [9–12]. *Anaplasma capra* is distributed all over the world, including China, France, Japan, South Korea, and Italy [5,7,13–15], posing a potential health threat to both humans and animals.

The Genus *Anaplasma* comprises a group of Gram-negative obligate intracellular bacteria consisting of many members, e.g. *A. bovis*, *A. ovis*, *A. marginale*, *A. centrale*, and *A. phagocytophilum* [16]*.* While *A. ovis*, *A. marginale,* and *A. centrale* are known as intraerythrocytic pathogens, *A. bovis* and *A. phagocytophilum* infect monocytes and neutrophil granulocytes, respectively [17,18]. *Anaplasma phagocytophilum* presents as a mild to severe febrile illness, including multiple organ failure and death [1,19–21]. *Anaplasma capra* is a novel zoonotic *Anaplasma* sp., included with other *Anaplasma* species in the list of prokaryotic names with standing in nomenclature (LPSN), but not validly published. Its morphological characteristics and type cells infected are unclear. Although molecular detection and identification based on *groEL*, 16S rRNA, *gltA*, *msp2,* and *msp4* genes of *A. capra* have been performed in the last five years [6], neither morulae nor other forms of the pathogen had been detected so far in peripheral blood smears [1,10]. Hence, studies to determine the host parasite site, morphological characteristics, and pathogenesis of *A. capra* is needed to distinguish it from other tick-borne *Anaplasma* infections.

## Material and methods

### Blood samples collection and preparation

Two goats with a history of tick bites were purchased from a goat farmer in Luoyang City, Henan province, central region of China. One goat was *A. capra*-positive by PCR, and the other was *A. capra*-negative. EDTA blood samples were collected from the two goats, and the erythrocytes and leukocytes separated from the blood samples using an Erythrocytes Separation Kit (TBD, Tianjin, China). The density gradient of the separation solution separates erythrocytes and leukocytes. Subsequently, DNA of the whole blood cells, erythrocytes and leukocytes, were extracted from the samples using a Blood DNA Kit (OMEGA, Norcross, GA, USA), according to the manufacturer’s recommendations. All DNA samples were stored at −20°C until used.

### PCR, gene sequencing and phylogenetic analysis

A multiplex PCR detection assay was used to detect erythrocyte and leukocyte DNA samples targeting the *A. capra groEL* gene, as previously described [22] (Table 1). *Anaplasma capra*-positive samples were genetically profiled by amplification of the16S rRNA (rrs) gene as well as *gltA* and *msp4* genes as previously described [1] (Table 1). Selected, *A. marginale*, *A. platys,* and *A. centrale* genes were used to exclude co-infections in *A. capra*-positive samples (Table 1). As positive controls, a co-infected DNA sample (concurrently infected with “*A. capra,*” *A. bovis, A. ovis,* and *A. phagocytophilum*) and *A. marginale-*, *A. platys-,* and *A. centrale*-positive samples were used, and negative control double distilled H_2_O was used.

**Table 1.** Primers used in the PCR assays of *Anaplasma* spp.

Pathogens	Target gene	Primer sequence (5′-3′)		Annealing temperature (°C)	Amplicon size (bp)	References
		Forward	Reverse			
*A. capra* sp. nov	16S rRNA	GCAAGTCGAACGGACCAAATCTGT	CCACGATTACTAGCGATTCCGACTTC	58	1261	[1]
	*gltA*	GCGATTTTAGAGTGYGGAGATTG	TACAATACCGGAGTAAAAGTCAA	55	1031	[1]
		TCATCTCCTGTTGCACGGTGCCC	CTCTGAATGAACATGCCCACCCT	60	594	
	*msp4*	GGGTTCTGATATGGCATCTTC	GGGAAATGTCCTTATAGGATTCG	53	656	[42]
	*groEL*	TGAAGAGCATCAAACCCGAAG	CTGCTCGTGATGCTATCGG	63^a^	874	[22]
*A. bovis*	*groEL*	GTGGGATGTACTGCTGGACC	ATGGGGAGAGATATCCGCGA		529	
*A. ovis*	*msp4*	TGAAGGGAGCGGGGTCATGGG	GAGTAATTGCAGCCAGGCACTCT		347	
*A. phagocytophilum*	16S rRNA	AGTGCTGAATGTGGGGATAATTTATCTCCGTG	CTAATCTCCATGTCAAGGAGTGGTAAGGTTT		172	
*A. marginale*	*msp4*	GGGAGCTCCTATGAATTACAGAGAATTGTTTAC	CCGGATCCTTAGCTGAACAGGAATCTTGC	62	867	[43]
*A. platys*	16S rRNA	AGTTTGATCATGGCTCAG	CCATGGCGTGACGGGCAGTGTG	58	1400	[44]
		GATTTTTGTCGTAGCTTGCTATG	TAGCACTCATCGTTTACAGC	56	678	
*A. centrale*	*groEL*	GCGCATTCTGGAGGCTG	GCGTTTGACTTGGCTGTGTC	64	1482	[45]

^a^ Annealing temperature for multiplex PCR.

The PCR products were sequenced to confirm the presence of *Anaplasma capra* DNA. The sequences obtained (GenBank Accession Nos.: *groEL* MT804297, 16S rRNA MT799937, *gltA* MT804296, *msp4* MT804298) were compared with published sequences in GenBank using BLASTn search (https://blast.ncbi.nlm.nih.gov/Blast.cgi). Phylogenetic analyses were performed and phylogenetic trees constructed based on the sequence distance method using the neighbor-joining (NJ) algorithm with Mega 7.0 software.

### Electron microscopy

*Anaplasma capra-*positive erythrocytes were fixed with a commercial electron microscope fixing solution (Solarbio, Beijing, China). After fixation, the cells were removed by centrifugation. The fixed cells were 0.01 M phosphate buffer solution (PBS) buffer-washed, post-fixed with 2% OsO_4_, water-washed, and dehydrated in graded ethanol (EtOH) series (30%, 50%, 70%, 80%, 90%, 100%, and 100% at 15-min intervals). For SEM, after the cells were dehydrated in graded EtOH series, the EtOH was replaced with isoamyl acetate, mounted on a metal stub, sputter-coated with gold for 5 min, and examined using a SU8100 electron microscope (HITACHI, Japan) at 3 kV. For TEM observation, the cells were infiltrated with acetone and Pon 812 epoxy resin (SPI, West Chester, USA) (1/1, 2 h; then, 1/2, 12 h) and cured at 60°C for 48 h. The cured resin blocks were trimmed, thin-sectioned, thin sections collected on formvar copper 200 mesh grids, and then post-stained with 2% aqueous uranyl acetate and Reynold’s lead citrate. The sections were examined using a HT7700 electron microscope (HITACHI, Japan) [23]. The morulae of *A. capra*, following lysis of the erythrocytes and centrifuged at 12,000 × g, were observed by TEM.

### Wright–Giemsa staining, CISH, immunocytochemistry, and IFA

Blood cell smears were fixed in methanol for 10 min and stained with Wright–Giemsa stain, and the intracellular morulae observed under light microscopy. To identify the specificity of the intracellular morulae detected in the blood cells, CISH, immunocytochemistry, and IFA were conducted with *A. capra*-positive samples.

CISH was performed with a commercial kit according to the manufacturer’s protocol (Invitrogen, Carlsbad, USA). Briefly, the blood films were reverse-stained with eosin, visualized using Digital Slice Scanner (Pannoramic MIDI, Hungary), and fixed with a fixative (Solarbio, Beijing, China). The CISH probes were designed specifically for *Anaplasma capra* (Invitrogen, Carlsbad, USA). To achieve sufficient signal to background ratio, multiple probes were targeted along each individual lncRNA/mRNA sequence of the *A. capra* sp. nov *groEL* gene (KM206275). A set of 16 probes covering the entire length of the RNA molecule allowed for optimal signal strength. The probes were labeled with digoxigenin (DIG), and brown-stained cells were considered positive.

For immunocytochemistry, the *A. capra*-positive serum samples collected from the goat were used as the primary antibody and incubated at 4°C overnight. Subsequently, HRP-conjugated Rabbit Anti-Goat IgG (H+L) (Servicebio, Wuhan, China) was applied to the smears and incubated at 37°C for 1 h. The antibody was visualized using 3,3′-diaminobenzidine (Servicebio, Wuhan, China), and the images recorded using a light microscope (Nikon, Tokyo, Japan). As negative controls, the antiserum from the *A. capra*-negative goat and *A. capra*-negative blood smears were processed in the same manner and examined.

For IFA, *A. capra*-positive whole blood cells were processed for the preparation of antigen slides. The *A. capra*-positive serum samples collected from goats were used as the primary antibody, and Donkey anti-Goat IgG (H+L) Cross-Adsorbed Secondary Antibody (Thermo Fisher Scientific, Carlsbad, USA) used as a secondary antibody [20]. The *A. capra*-negative whole blood cells smear were used as negative controls. The slides were examined using a Digital Slice Scanner (Pannoramic MIDI, Hungary) and cells with blue fluorescence considered positive.

## Results

**Figure 1.**
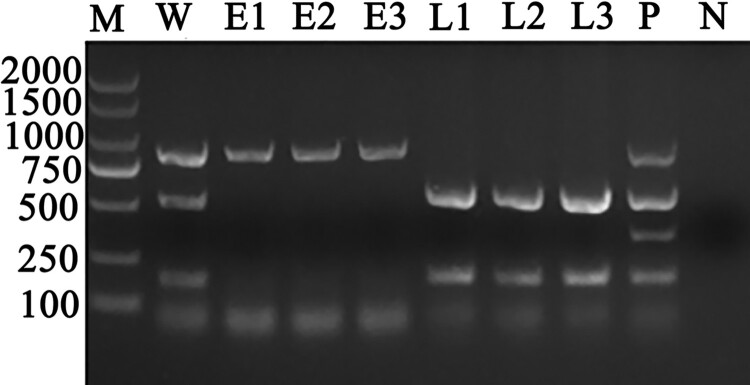
Results of multiplex PCR detection assay for the identification of *A. capra* sp. nov in whole blood, erythrocyte, and leukocyte DNA samples from the same goat whole blood sample. Lane W: Whole blood DNA sample positive for *A. capra* sp. nov (874 bp), *A. bovis* (529 bp), and *A. phagocytophilum* (172 bp). Lanes E1–E3: Erythrocytes DNA samples positive for *A. capra* sp. nov (874 bp). Lanes L1–L3: Leukocytes DNA samples positive for *A. bovis* (529 bp) and *A. phagocytophilum* (172 bp). Lane P: Positive control of multiplex PCR for detecting *A. capra* sp. nov, *A. bovis*, *A. ovis* (347 bp), and *A. phagocytophilum*. Lane N: Negative control.

**Figure 2.**
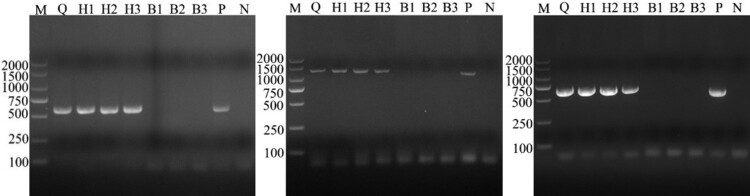
Identification results of different blood cell DNA samples by PCR based on gltA, 16S rRNA and msp4 genes of *A. capra*. A-C was the result based on gltA, 16S rRNA and msp4 gene, respectively. Erythrocyte DNA samples were *A. capra*-positive only based on multiplex loci, while leukocytes DNA samples were *A. capra*-negative.

The multiplex PCR detection assay showed that the whole blood DNA samples were positive for “*A. capra,*” *A. bovis,* and *A. phagocytophilum*. Three erythrocyte DNA samples were *A. capra*-positive only, while three leukocytes DNA samples were both *A. bovis-* and *A. phagocytophilum*-positive (Figure 1). Therefore, multi-site identification was conducted based on 16S rRNA, *gltA,* and *msp4* genes of *A. capra* using erythrocyte and leukocyte DNA samples (Figure 2). The PCR identification results based on multiple gene loci showed that all erythrocyte DNA samples were *A. capra*-positive and *A. marginale*-, *A. platys*-*,* and *A. centrale*-negative; leukocyte DNA samples were *A. capra*-negative; and samples infected with *A. bovis*, *A. ovis*, *A. marginale*, *A. platys,* and *A. centrale* were *A. capra*-negative.

**Figure 3.**
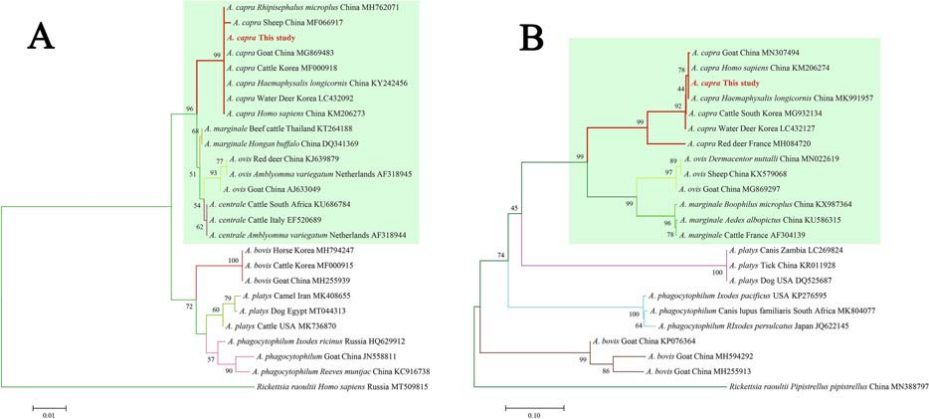
Phylogenetic analysis of *A. capra* sp. nov identified in this study based on 16S rRNA (1261 bp, A) and *gltA* (594 bp, B) genes. The trees were constructed using NJ method with MEGA 7.0 software and the numbers on the tree indicate bootstrap values for the branch points. Bootstrap analysis was performed with 1000 replicates. Numbers on the branches indicate percent support for each clade. Red font denotes the sequences obtained in this study. *Rickettsia raoultii* was used as outgroup. The green background represents intraerythrocytic *Anaplasma* spp.

**Figure 4.**
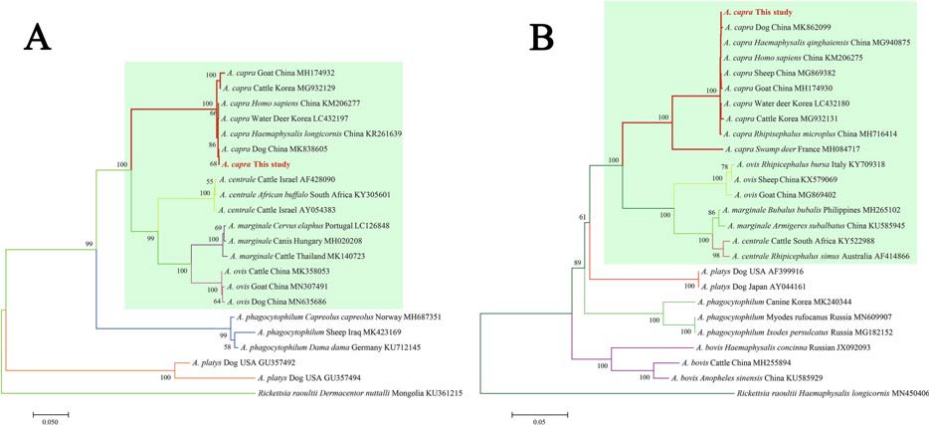
Phylogenetic analysis of *A. capra* sp. nov identified in this study based on *msp4* (656 bp, A) and *groEL* (874 bp, B) genes. The trees were constructed using NJ method with MEGA 7.0 software and the numbers on the tree indicate bootstrap values for the branch points. Bootstrap analysis was performed with 1000 replicates. Numbers on the branches indicate percent support for each clade. Red font denotes the sequences obtained in this study. *Rickettsia raoultii* was used as outgroup. The green background represents intraerythrocytic *Anaplasma* spp.

Subsequently, the sequences of *groEL* (874 bp), 16S rRNA (1261 bp), *gltA* (594 bp), and *msp4* (656 bp) of *A. capra* were obtained and submitted to GenBank (Accession Nos. MT804297, MT799937, MT804296, and MT804298, respectively). The entire 16S rRNA sequence of the *Anaplasma* sp. from a goat was 100% homologous to sequences observed in a human (GenBank Accession No. KM206273) and *H. longicornis* (GenBank Accession No. KY242456). The phylogenetic tree, based on 16S rRNA sequences, showed that the isolated *Anaplasma* sp. nov is in the same clade with *A. capra* from *R. microplus* (MH762071), *H. longicornis* (KY242456), *H. sapiens* (KM206273), goat (MG869483), sheep (MF066917), cattle (MF000918), and Korean water deer (LC432092), but clearly separated from other *Anaplasma* spp. (Figure 3(A)). Further analyses of *gltA*, *msp4,* and *groEL* sequences showed that *Anaplasma* sp. nov demonstrated a high homology to *A. capra* reported previously (KM20627, KM206274, KM206275, KM206277) (Figures 3 and 4(A,B)). Phylogenetic tree analysis based on the sequences of *gltA*, *msp4,* and *groEL* genes indicated that the *Anaplasma* sp. sequence detected in the present study is in the same clade with *A. capra* sequences previously reported (Figures 3 and 4(B)). However, the sequence from the French red deer and swamp deer appeared to have a different genotype, which was in the same cluster with “*A. capra*,” but on a different branch (Figures 3 and 4(B)). Moreover, phylogenetic trees based on the four gene sequences showed that *A. capra* is in the same cluster with *A. marginale*, *A. centrale,* and *A. ovis,* suggesting that *A. capra* and the three *Anaplasma* spp. may have some similar characteristics.

**Figure 5.**
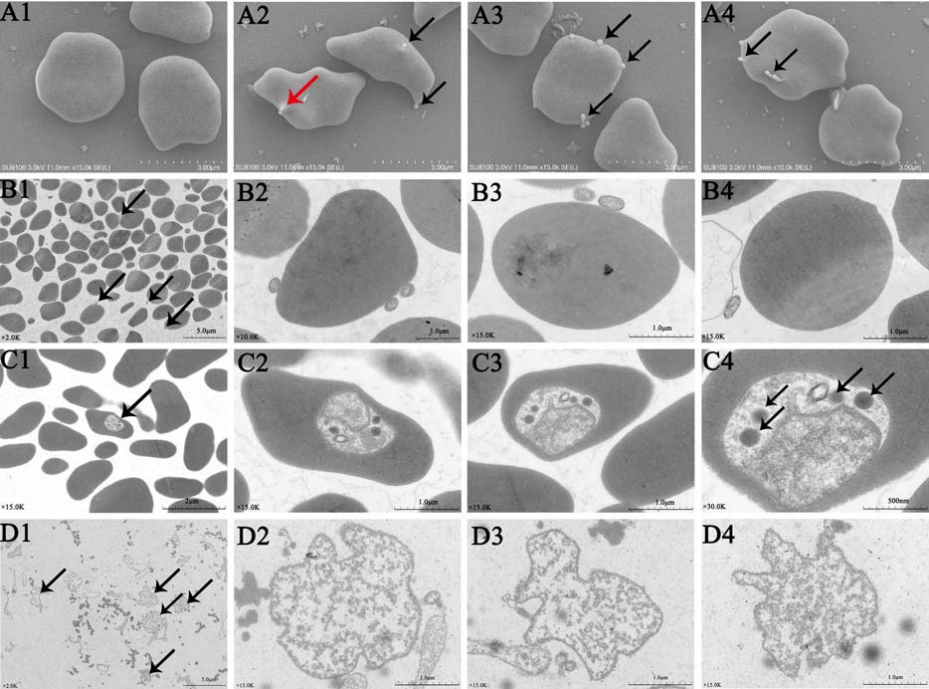
Photomicrographs of *A. capra* sp. nov and infected goat erythrocytes. A1–A4, SEM photomicrographs of erythrocytes and *A. capra* sp. nov. Normoerythrocytes (A1) and infected erythrocytes*.* Arrows indicate *A. capra* sp. nov (A2–A4). A2 shows invading pathogen (red arrowhead). B1–B4 and C1–C4, TEM photomicrographs of erythrocytes and *A. capra* sp. nov. Uranyl acetate and Reynold's lead citrate stain. Morulae are observed beside and within multiple erythrocytes (arrows). B2–B4 and C2–C4 show higher magnification of the morulae. Electron-dense particles (Lysosomes, arrows) are observed in less-dense areas (C2–C4, arrows). D1–D4, TEM photomicrographs of the separated *A. capra* sp. nov morulae. D1 (arrows) shows lower magnification of the morulae and D2–D4 present higher magnification of the morulae. No Lysosomes are observed in the morulae.

The erythrocytes infected with *A. capra* were examined using electron microscopy. SEM images demonstrated the presence of one or more *A. capra* cells (0.2–0.4 µm in diameter) on the outer surface of the erythrocytes (Figure 5(A2–A4)). Additionally, invasion of the erythrocytes was also observed (Figure 5(A2)). TEM images showed one or more typical small round to oval morulae (0.2 × 0.4 µm) on the membrane and in multiple erythrocytes (Figure 5(B1–B4, C1–C4)). The morulae in the cytoplasm of the erythrocytes were about 10 times (0.8 × 1 µm) the size of those on the outside cells (Figure 5(C1–C4)). The morulae density was not uniform, and electron-dense particles (Lysosomes) were observed in less-dense areas of the pathogen (Figure 5(C2–C4)). When separated from erythrocytes, the morulae consisted of a membrane-bound vacuole containing dense granular bacterial subunits with a lower pathogen density than intracellular morulae, and the lysosomes were absent (Figure 5(D1–D4)).

**Figure 6.**
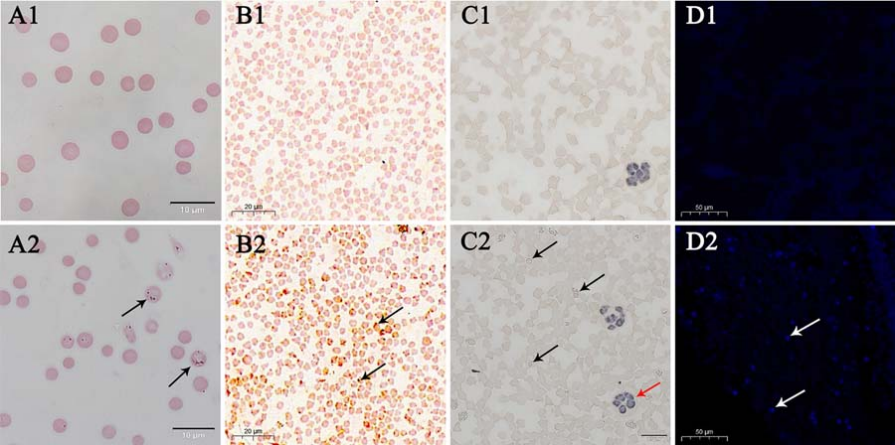
Wright–Giemsa, CISH, immunocytochemistry, and IFA analyses of uninfected and infected goat erythrocytes. A1–A2, Wright–Giemsa-stained erythrocytes. B1–B2, CISH assay of the erythrocytes smear. A1–D1 are negative controls. The probe was labeled with DIG. C1–C2 and D1–D2, immunocytochemistry and IFA of erythrocytes incubated with positive goat serum. Black and white arrows denote *A. capra* sp. nov-positive erythrocytes. Red arrows show *A. phagocytophilum*-positive neutrophilic granulocytes.

Wright–Giemsa-stained *A. capra*-positive erythrocytes smear showed numerous small morulae in the cytoplasm (Figure 6(A1–A2)). In the CISH assay, microscopic observation demonstrated reddish-brown (DIG) specific probe signals on the surface and inside of *A. capra*-positive erythrocytes (Figure 6(B1)). The negative controls showed erythrocytes with non-specific probe signals (Figure 6(B2)).

Immunocytochemistry-positive cells were observed in erythrocytes, and appeared as intracytoplasmic aggregates, known as morulae (Figure 6(C1–C2)). No morulae were observed in the negative controls. In addition, immunocytochemistry-positive cells were occasionally noted in neutrophilic granulocytes, as the whole blood cells were also *Anaplasma phagocytophilum*-positive.

IFA positive fluorescence was obtained with *A. capra* antiserum, confirming that the cells contained *A. capra* parasites (Figure 6(D2)). In contrast, negative-control erythrocytes showed no fluorescence (Figure 6(D1)). Thus, owing to its unique morphological characteristics and parasitic site, we propose to revise the *Anaplasma* genus and formally name “*A. capra*” as *Anaplasma capra* sp. nov.

## Discussion

*Anaplasma capra* sp. nov was first identified from seven goats based on the 16S rRNA gene in Southwest China. Sequences from the goats formed two *Anaplasma* spp. clusters that clearly distinguished them from the clusters of *A. marginale*, *A. centrale,* and *A. ovis*, suggesting that this pathogen could be a potential new *Anaplasma* sp. [2]. Similarly, Liu et al. [17] (2012) performed molecular analysis of the pathogen based on 16S rRNA gene in goats from Zhejiang, China. A comparable genotype of this *Anaplasma* sp. was detected in humans with tick-bite history since 2015 based on *groEL*, 16S rRNA, *gltA*, *msp2,* and *msp4* genes [1], and was provisionally nominated as “*A. capra,*” belonging to the Genus *Anaplasma*. Likewise, we found that *A. capra* sp. nov exhibited significant differences in *groEL*, 16S rRNA, *gltA,* and *msp4* genes, when compared with other *Anaplasma* spp. [5]. Many studies have used this molecular method to identify and characterize *A. capra* sp. nov in different hosts [6,7,24–26].

*Anaplasma capra* sp*.* nov appears to exhibit at least two different genotypes, both are likely zoonotic [4, 7]. The sequences of *gltA* and *groEL* genes examined in the present study formed two clusters in the phylogenetic tree. In a previous study, sequence analysis based on *gltA* gene demonstrated a novel *A. capra* sp*.* nov genotype in sheep, which was distinct from the isolates identified from patients in northeastern China [27]. Furthermore, our previous study also identified two genotypes based on *gltA* gene of *A. capra* sp. nov in sheep and goats from Henan, China [5]. Additionally, it has been reported that sequences based on the 16S rRNA gene from goat strains and the *gltA* gene from sheep strains formed two distinct *A. capra* sp. nov clusters [2,28]. Hence, our future research will involve genotyping *A. capra* sp. nov. As both the genotypes of *A. capra* sp. nov have been detected in ruminants and hard ticks, they pose a potential health threat to humans. The host range of the zoonotic genotypes needs further study, especially in areas infested with hard ticks.

*Anaplasma* spp*.* that have been reported to infect erythrocytes include *Anaplasma marginale*, *A. centrale,* and *A. ovis* [29–31]*. A. marginale* and *A. centrale* were first identified in 1910 and 1911, respectively, by Theiler, who observed small morulae of *A. marginale* that was previously mistakenly as *Babesia bigemina* at the periphery of stained cattle erythrocytes [18,32]. *Anaplasma centrale* forms smaller and more central morulae and is closely associated with *A. marginale* within infected erythrocytes [30], while *A. ovis* was first detected in the central area of sheep erythrocytes in 1912 [31]. However, there are still no relevant reports on the specific site location of *A. capra* sp. nov*.* Similar to other *Anaplasma* spp., the target cells of this pathogen may be one type of blood cell. Li et al. presumed that *A. capra* sp. nov most probably infect erythrocytes or endothelial cells in mammals; however, this assumption has not yet been proved [1]. Interestingly, in the present study, *A. capra* sp. nov was detected inside or at the periphery of erythrocytes that were *A. capra* sp. nov PCR-positive and *A. marginale*, *A. centrale,* and *A. ovis* PCR-negative, and could infect human erythrocytes. The morphological characteristics of *A. capra* sp. nov determined in the present study are consistent with those previously reported [1], and different from the other intraerythrocytic *Anaplasma* spp. [20,23,33–35]. These findings significantly contribute to the research on this novel zoonotic *Anaplasma* sp.

Erythrocytes infected with intraerythrocytic *Anaplasma* spp. are destroyed by macrophages, resulting in mild to severe hemolytic anemia [18]. Sheep and goats infected with *Anaplasma* spp. develop a mild to severe disease, with clinical symptoms such as fever, pale mucous membranes, weight loss, icterus, anorexia, depression, lower milk production, coughing, dyspnea, gastrointestinal signs, abortion, and death [36,37]. Humans infected with *A. capra* sp. nov exhibit fever, headache, malaise, dizziness, chills, nausea, vomiting, diarrhea, and laboratory abnormalities, e.g. high hepatic aminotransferase concentrations, leucopenia, and thrombocytopenia [1]. The pathogen is carried and may be transmitted to their zoonotic and domestic hosts by many species of hard ticks that are widely distributed [9–12,38–41]. Thus, *A. capra* sp. nov is a substantial threat to humans and domestic animals, and precautions should be taken to prevent anaplasmosis caused by this novel tick-borne pathogen.

## Conclusion

To our knowledge, this was the first study to detect a novel zoonotic tick-borne *Anaplasma* sp., *A. capra*, in host erythrocytes. The molecular characteristics, morphological features, and parasitic sites of *A. capra* were noted to differ from those of other *Anaplasma* spp. Hence, we propose revising the Genus *Anaplasma* and formally naming “*A. capra*” as *A. capra* sp. nov.
